# Identification and characterization of inorganic-phosphate-solubilizing bacteria from agricultural fields with a rapid isolation method

**DOI:** 10.1186/s13568-018-0575-6

**Published:** 2018-03-27

**Authors:** Bang-Xiao Zheng, Muhammad Ibrahim, Ding-Peng Zhang, Qing-Fang Bi, Hong-Zhe Li, Guo-Wei Zhou, Kai Ding, Josep Peñuelas, Yong-Guan Zhu, Xiao-Ru Yang

**Affiliations:** 1 Key Laboratory of Urban Environment and Health, Institute of Urban Environment, Chinese Academy of Sciences, Xiamen, 361021 People’s Republic of China; 2 University of Chinese Academy of Sciences, Beijing, 100049 People’s Republic of China; 3 Consejo Superior de Investigaciones Científicas (CSIC), Global Ecology Unit, Centre for Ecological Research and Forestry Applications (CREAF), Universitat Autònoma de Barcelona (UAB), Bellaterra, 08193 Barcelona, Catalonia Spain; 4 CREAF, Cerdanyola del Vallès, 08193 Barcelona, Catalonia Spain; 5 State Key Laboratory of Biocontrol, Key Laboratory of Biodiversity Dynamics and Conservation of Guangdong Higher Education Institutes, College of Ecology and Evolution, Sun Yat-sen University, Guangzhou, 510275 People’s Republic of China; 6 MOE Key Laboratory of Environment Remediation and Ecological Health, College of Environmental and Resource Sciences, Zhejiang University, Hangzhou, 310058 People’s Republic of China; 7 State Key Laboratory of Urban and Regional Ecology, Research Center for Eco-Environmental Sciences, Chinese Academy of Sciences, Beijing, 100085 China

**Keywords:** Phosphorus, Inorganic phosphate solubilizing bacteria, Isolation, Characterization

## Abstract

The ability to solubilize fixed inorganic phosphorus (P) for plant growth is important for increasing crop yield. More P can be released by inoculating soil with inorganic-phosphate-solubilizing bacteria (iPSBs). We used 96-well microplates instead of traditional 200-mm petri dishes to rapidly screen iPSB strains for their solubilizing ability. We simultaneously obtained 76 iPSB isolates from 576 wells containing two agricultural soils. This method conveniently identified positive iPSB strains and effectively prevented fungal cross-contamination. Maximum-likelihood phylogenetic trees of the isolated strains showed that *Bacillus megaterium* was the most dominant iPSB, and strains Y99, Y95, Y924 and Y1412 were selected as representatives for the analysis of P solubilization. Succinic acid was the main organic acid of *B. megaterium* for releasing P. It was strongly correlated with the increase in soluble P concentration during 168 h of incubation of these four strains. pH was negatively exponentially correlated with the amount of soluble P in the medium, and the amount of succinic acid was strongly linearly correlated with the amount of P released (*P* < 0.001), suggesting that organic acid may mobilize microbial P. Our study provides an efficient and effective method for identifying and analyzing the growth of iPSB strains able to solubilize inorganic P and gives a better understanding of the mechanism of P solubilization.

## Introduction

Phosphorus (P), a non-renewable macronutrient, plays an essential role in plants (Elser et al. 2007). Inorganic P is mined to produce chemical P fertilizers that are extensively applied to cropland (Elser and Bennett 2011; Penuelas et al. 2013). The majority of soluble inorganic P, however, is rapidly immobilized by soil fixation and becomes unavailable for plant uptake, leading to low P-use efficiency and potentially excess P (Kochian 2012). Soil P must thus be managed to minimize its loss and increase its use efficiency.

Microorganisms are actively involved in many biogeochemical processes, including the mineralization, solubilization and transformation of soil P (van der Heijden et al. 2008). Inorganic-phosphate-solubilizing bacteria (iPSBs) are particularly effective in releasing P from pools of inorganic P. iPSBs can also prevent the liberated P from being fixed again (Richardson et al. 2009; Richardson and Simpson 2011). Screening highly efficient iPSBs as soil inoculum is a useful method for improving plant growth and yield (Richardson et al. 2009). Many methods for screening iPSBs have been reported (Chen et al. 2006; Chung et al. 2005; Mehta and Nautiyal 2001; Nautiyal 1999), but isolating iPSBs on separate petri dishes can be time-consuming and labor-intensive.

The mechanism of inorganic-P microbial mobilization is generally associated with extrusion of low-molecular-weight organic acids (Goldstein 1995), which can competitively chelate the cations bound to P via hydroxyl and carboxyl groups and convert them into soluble forms (Jones and Oburger 2011; Richardson and Simpson 2011). The solubilization of inorganic P, however, is complex and depends on numerous factors such as soil properties, plant nutritional requirements and physiological and growth conditions. Studies of the factors affecting solubilization are thus still needed.

We developed a rapid method for screening iPSBs and evaluating their effectiveness at solubilizing inorganic P, with an emphasis on potential highly efficient iPSBs for agricultural use. The isolated iPSB strains were identified and characterized. The types of organic acids secreted by the iPSB strains with high P-solubilizing abilities and the relationships between the organic acids, pH and P solubilization were analyzed.

## Materials and methods

### Soil sampling and characterization

Soil samples were collected from agricultural fields near Hailun in Heilongjiang Province (47′26″N, 126′38″E) and Yingtan in Jiangxi Province (28′14″N 116′54″E), China (Table 1). Approximately 500 g of surface soil (0–15 cm) was collected after crop harvests in June 2014. The soils were then air-dried, sieved (0.2 mm) and stored at 4 °C until analysis.

**Table 1:** Basic information and soil properties of the two soil samples

Sample	Location	pH	Total P (g kg^−1^)	Olsen P (mg kg^−1^)	Organic P (mg kg^−1^)	Inorganic P (mg kg^−1^)
Hailun	47′26″N, 126′38″E	5.70 ± 0.08	779.35 ± 44.33	58.80 ± 2.64	351.15 ± 6.37	428.21 ± 37.96
Yingtan	28′14″N 116′54″E	5.01 ± 0.03	522.60 ± 7.54	23.56 ± 1.53	370.57 ± 5.50	152.04 ± 2.05

Soil pH was measured using a 1:2.5 (w/v) suspension of dry soil: water and a XL60 pH meter (Fisher Scientific, USA) (Shen et al. 2008). The amounts of total P and available P (Olsen P) were determined using the molybdate-blue method (Murphy and Riley 1962) and sodium bicarbonate extraction (Olsen et al. 1954) after acid digestion (Parkinson and Allen 1975), respectively. The inorganic-P concentration was measured by shaking 0.2 g of soil in 20 mL of 1 M HCl at 200 rpm for 30 min, followed by centrifugation at 4200*g* for 10 min. The amount of inorganic P in the supernatant was then measured by the molybdate-blue method. The amount of organic P was calculated by subtracting the inorganic-P concentration from the amount of total P.

### Rapid screening of iPSB strains

**Fig. 1:**
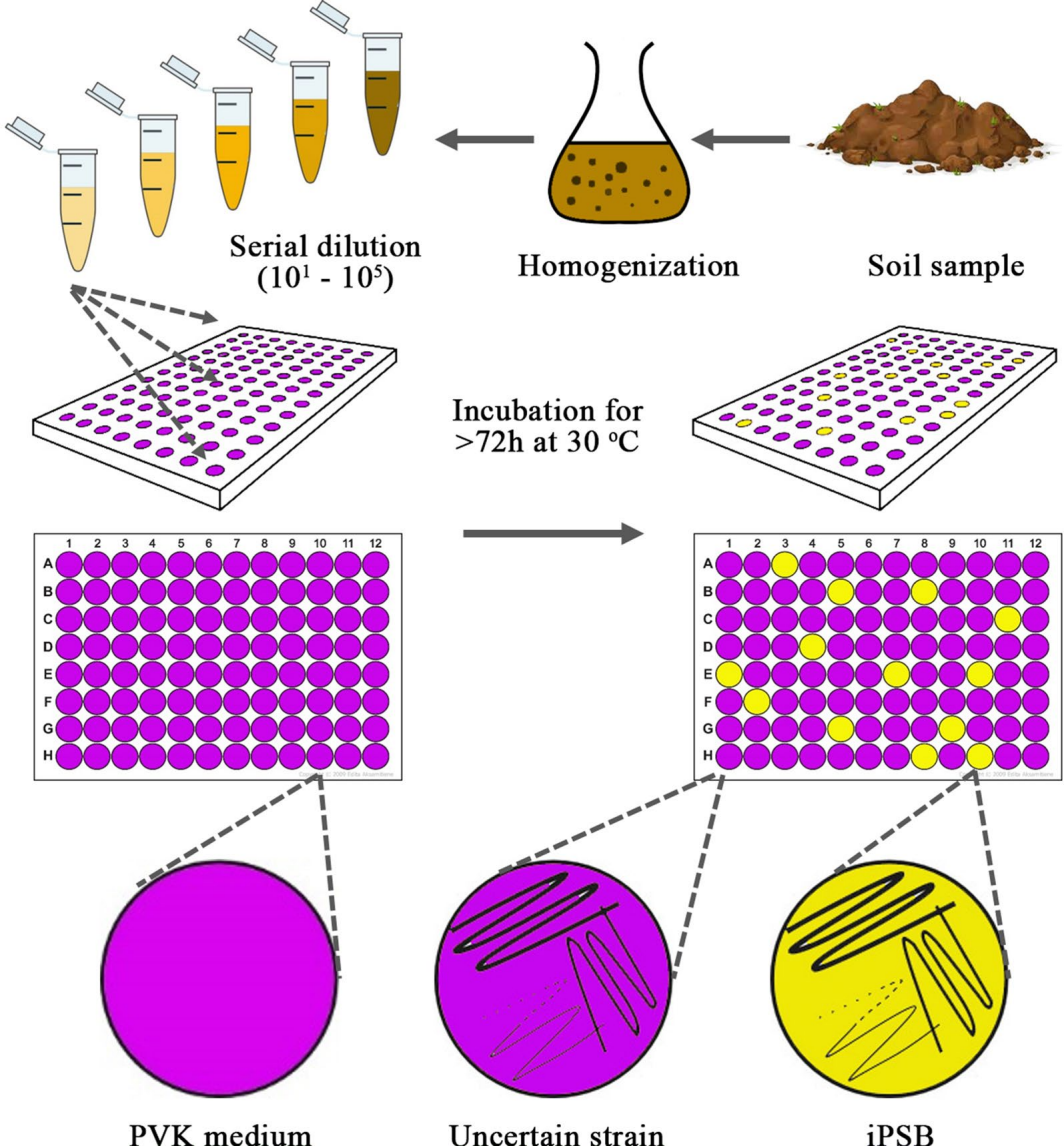
Flowchart of the 96-well iPSB screening method. The sampled soil was homogenized in autoclaved water and serially diluted for incubation at 30 °C for > 72 h in 96-well microplates containing PVK with bromocresol purple as an indicator. Wells with no color change or strain growth, or with strain growth but no color change, were classified as containing unknown strains without P-solubilizing ability. Wells that produced a yellow color were classified as containing iPSB strains

The iPSBs were rapidly screened using 96-well microplates (Fig. 1). A modified Pikovskaya medium (PVK) without yeast extract (Nautiyal 1999) was used as the culture medium and was added to each well in advance. The PVK was supplemented with 10 μM bromocresol purple as an indicator. For each microplate, 1 g of soil was homogenized with 100 mL of sterilized water, and this suspension was then serially diluted (10–10^5^). One microliter of diluted soil suspension was added to each well and then incubated at 30 °C for at least 72 h. An uninoculated well served as a control. Wells with no bacterial growth were considered negative. Wells with bacterial growth but no obvious color change were classified as containing uncertain strains, and wells with obvious bacterial growth and a yellow color were classified as positive. Three replicates of each dilution were tested. The effectiveness of this method was verified by streaking both uncertain and positive strains on plates containing solid modified PVK.

### Biochemical characterization of the iPSB strains

The pH and soluble-P concentration of the medium after incubation were used as indices for iPSB screening. The indices were measured by incubating all strains in 50 mL of liquid modified PVK (without agar and indicator) at 30 °C for 72 h. The supernatants obtained after centrifugation (4200*g* for 10 min) were used to measure pH with a XL60 pH meter (Fisher Scientific, USA) and phosphate concentration using the molybdate-blue method (Murphy and Riley 1962).

### Phylogenetic identification with 16S rRNA sequencing

All positive strains were incubated in liquid modified PVK (without indicator) at 30 °C for 24 h. The full lengths (~ 1500 bp) of 16S rRNA genes were obtained by PCR using universal bacterial primers (24F: AGAGTTTGATCCTGGCTCAG and 1492R: TACGGYTACCTTGTTACGACTT) (Farris and Olson 2007). Each 50-μL PCR reaction contained 1 μL of *Premix Ex Taq* Hot Start Version (TAKARA, Dalian, China), 0.2 μM each primer and 1 μL of bacterial culture suspension as DNA template. The amplification protocol was an initial denaturation at 95 °C for 4 min, 30 cycles of 95 °C for 30 s, 58 °C for 90 s and 72 °C for 30 s and a final 5-min extension at 72 °C. The amplicons were purified with a Universal DNA Purification Kit (TIANGEN, Beijing, China) and submitted for sequencing (Invitrogen, Shanghai, China). The sequences were aligned with those from bacterial lineages in GenBank at the National Center for Biotechnology Information (NCBI) (http://www.ncbi.nlm.nih.gov/) with the BLAST program. The 16S rRNA sequences of all strains were uploaded to the NCBI Sequence Read Archive with Accession Numbers KU647195-KU647270 (Table 2).

**Table 2:** Accession numbers, medium pH and soluble-P concentrations of the iPSB strains after incubation for 72 h, and the closest reference strains

Strain	Accession number	pH of medium	Soluble-P concentration (μg mL^−1^)	Closest reference strain		
				Affiliation	Accession number	Similarity (%)
*Bacillus megaterium* 01-A3	KU647195	4.80	85.57	*Bacillus* sp. BS3(2015)	KR063183	99
*Bacillus megaterium* 02-A7	KU647196	4.59	89.08	*Bacillus* sp. KU6	JF895481	99
*Pseudomonas frederiksbergensis* 03-D2	KU647197	5.21	64.28	*Pseudomonas* sp. WS06	JN210901	99
*Rhodococcus opacus* 04-OD7	KU647198	5.17	28.06	*Rhodococcus opacus* DSM 43205	LN827919	99
*Arthrobacter phenanthrenivorans* 05-OD11	KU647199	5.89	12.24	*Arthrobacter phenanthrenivorans* L43	LN890039	99
*Arthrobacter defluvii* 06-OD12	KU647200	8.34	59.11	Uncultured bacterium D1-57	KC554872	99
*Arthrobacter chlorophenolicus* 07-OD13	KU647201	5.58	20.84	*Arthrobacter* sp. M29	KF430812	99
*Arthrobacter oxydans* 08-OY2	KU647202	6.64	3.85	Uncultured bacterium D1-57	KC554872	99
*Arthrobacter* sp. 09-OY5	KU647203	5.11	43.00	*Arthrobacter* sp. WS03	JN210899	99
*Bacillus megaterium* 10-Y11	KU647204	4.77	106.46	*Bacillus megaterium* HNS88	KF933685	99
*Pseudomonas frederiksbergensis* 11-D3	KU647205	5.25	81.76	*Pseudomonas* sp. B3039	KC236870	99
*Massilia putida* 12-OD1	KU647206	4.63	97.29	Uncultured bacterium clone HF31	KR188907	99
*Duganella* sp. 13-D4	KU647207	5.69	10.78	*Duganella* sp. ZLP-XI	KF896136	99
*Bacillus megaterium* 14-Y2	KU647208	4.75	101.58	*Bacillus megaterium* Y20	JQ798391	99
*Pseudoduganella* sp. 15-Y6	KU647209	5.29	49.64	*Pseudoduganella* sp. NI28	KM087999	99
*Bacillus megaterium* 16-Y9	KU647210	4.66	80.20	*Bacillus* sp. RBB1	GU979225	99
*Bacillus megaterium* 17-Y5	KU647211	4.85	80.39	Uncultured *Bacillus* sp. clone T7F50d237	JN187411	99
*Variovorax paradoxus* 19-D4	KU647212	5.42	55.69	*Variovorax paradoxus* EPS	NR_074646	99
*Rhizobium leguminosarum* 20-OD2	KU647213	5.69	10.78	*Rhizobium* sp. SG6	LC042447	99
*Rhodanobacter* sp. 21-Y7	KU647214	7.72	2.58	*Rhodanobacter* sp. GR14-4	FJ821729	99
*Bacillus megaterium* 22-A1	KU647215	5.00	100.51	*Bacillus* sp. B2(2010b)	HM104462	99
*Pseudomonas frederiksbergensis* 23-D2	KU647216	5.20	63.41	*Pseudomonas* sp. WS06	JN210901	99
*Bacillus megaterium* 24-Y916	KU647217	4.79	109.39	*Bacillus* sp. BDH23	KF933618	99
*Rhodanobacter* sp. 25-Y8	KU647218	4.82	18.20	*Rhodanobacter* sp. GR14-4	FJ821729	99
*Bacillus megaterium* 26-Y91	KU647219	4.63	46.61	*Bacillus megaterium* HNS79	KF933676	99
*Bacillus megaterium* 27-Y93	KU647220	4.37	117.30	*Bacillus* sp. NyZ44	HQ231223	99
*Bacillus megaterium* 28-Y911	KU647221	4.54	126.48	*Bacillus megaterium* Bacteria I	KT427436	99
*Bacillus megaterium* 29-Y924	KU647222	4.55	136.83	*Bacillus* sp. BDH4	KF933626	99
*Bacillus megaterium* 30-Y1411	KU647223	4.48	134.39	*Bacillus* sp. WXGRY7	KJ184905	99
*Bacillus megaterium* 31-Y142	KU647224	4.71	97.29	*Bacillus megaterium* Bacteria I	KT427436	99
*Arthrobacter* sp. 32-OD9	KU647225	5.31	43.19	*Arthrobacter* sp. WS20	JN899573	99
*Streptomyces tumescens* 33-X1	KU647226	8.02	2.77	*Streptomyces tumescens* OTP-4-2	AF346485	99
*Streptomyces prasinopilosus* 34-Y1	KU647227	7.75	3.07	*Streptomyces* sp. GS15	JX679244	99
*Streptomyces rishiriensis* 35-Y3	KU647228	5.76	44.37	*Streptomyces* sp. YRA147	JX430828	99
*Kurthia zopfii* 36-Y7	KU647229	4.52	81.57	*Bacillus thermophilus* SgZ-10	NR_109677	97
*Rhodanobacter* sp. 37-Y8	KU647230	4.88	32.55	*Rhodanobacter* sp. GR14-4	FJ821729	99
*Bacillus megaterium* 38-Y92	KU647231	4.51	91.04	*Bacillus megaterium* HNS88	KF933685	99
*Bacillus megaterium* 39-Y94	KU647232	4.43	91.62	*Bacillus megaterium* HNS79	KF933676	99
*Bacillus megaterium* 40-Y95	KU647233	4.44	134.49	*Bacillus* sp. S10	HE662645	99
*Bacillus megaterium* 41-Y99	KU647234	4.41	159.48	*Bacillus megaterium* BS17	KR063197	99
*Bacillus megaterium* 42-Y910	KU647235	4.58	75.22	*Bacillus megaterium* B2	KT307979	99
*Bacillus megaterium* 43-Y912	KU647236	4.58	72.39	*Bacillus* sp. BDH23	KF933618	99
*Bacillus megaterium* 44-Y913	KU647237	4.50	46.51	*Bacillus megaterium* HNS88	KF933685	99
*Bacillus megaterium* 45-Y914	KU647238	4.65	94.26	*Bacillus megaterium* ML482	KC692173	99
*Bacillus megaterium* 46-Y923	KU647239	4.62	81.57	*Bacillus* sp. BDH4	KF933626	99
*Bacillus megaterium* 47-Y141	KU647240	4.62	70.73	*Bacillus* sp. BSp-2	KF835394	99
*Rhizobium* sp. 48-Y930	KU647241	7.86	3.75	*Rhizobium* sp. CC-SKC2	HQ113369	99
*Bacillus megaterium* 49-Y1412	KU647242	4.60	138.68	*Bacillus megaterium* HNS88	KF933685	99
*Rhizobium* sp. 50-Y1414	KU647243	8.02	5.60	*Rhizobium* sp. CC-SKC2	HQ113369	99
*Burkholderia cepacia* 51-Y1415	KU647244	5.00	2.03	*Burkholderia* sp. xin-1	KF059269	99
*Arthrobacter defluvii* 52-OD12	KU647245	4.59	76.10	Uncultured bacterium D1-57	KC554872	99
*Bacillus acidiceler* 53-Q11	KU647246	4.39	127.07	*Bacillus* sp. S21001	D84560	99
*Streptomyces prasinopilosus* 54-Y1	KU647247	5.29	49.64	*Streptomyces* sp. GS15	JX679244	99
*Pseudomonas frederiksbergensis* 55-D3	KU647248	4.96	35.87	*Pseudomonas* sp. B3039	KC236870	99
*Burkholderia phytofirmans* 56-OY3	KU647249	8.20	3.85	*Burkholderia* sp. C2-14	JF900054	99
*Variovorax paradoxus* 57-Y925	KU647250	5.30	10.88	*Variovorax* sp. LZA10	GQ861460	99
*Telluria mixta* 58-Y97	KU647251	4.62	106.85	Uncultured bacterium SuR5	AB608684	99
*Sphingomonas koreensis* 59-Y96	KU647252	7.31	2.77	Uncultured *Sphingomonas* sp. Plot4-G09	EU449628	99
*Streptomyces flaveolus* 60-OD3	KU647253	7.95	2.19	*Streptomyces flaveolus* NRRL B-1334	NR_116094	99
*Rhodanobacter* sp. 61-Y8	KU647254	4.49	62.23	*Rhodanobacter* sp. GR14-4	FJ821729	99
*Streptomyces* sp. 62-Y930	KU647255	6.50	3.46	*Streptomyces* sp. N4-145	EF063495	99
*Rhodococcus cercidiphylli* 63-OD5	KU647256	6.77	3.07	*Rhodococcus* sp. AB73	KC019201	98
*Bacillus megaterium* 64-Y98	KU647257	4.53	107.44	*Bacillus* sp. BDH23	KF933618	100
*Bacillus megaterium* 65-Y918	KU647258	4.71	69.75	*Bacillus megaterium* B2	KT307979	99
*Bacillus megaterium* 66-Y143	KU647259	4.55	82.84	*Bacillus megaterium* Bacteria I	KT427436	99
*Rhodococcus* sp. 67-OD10	KU647260	5.45	52.67	Uncultured bacterium clone Md-133	KT905708	99
*Arthrobacter oxydans* 68-OY1	KU647261	6.15	16.44	*Arthrobacter oxydans* BGSLP35	KP192013	99
*Pseudomonas* sp. 69-Y94	KU647262	4.87	71.51	*Pseudomonas* sp. B3042	KC236872	99
*Bacillus megaterium* 70-Y917	KU647263	4.43	76.10	*Bacillus* sp. S10	HE662645	99
*Pseudomonas* sp. 71-Y928	KU647264	5.41	37.82	*Pseudomonas* sp. B3042	KC236872	99
*Bacillus megaterium* 72-Y13	KU647265	4.61	112.03	*Bacillus megaterium* HNS79	KF933676	99
*Bacillus megaterium* 73-Y142	KU647266	4.77	106.46	*Bacillus megaterium* HNS88	KF933685	99
*Streptomyces* sp. 74-Y144	KU647267	5.00	22.50	*Streptomyces* sp. N4-145	EF063495	99
*Leifsonia shinshuensis* 75-Y145	KU647268	4.54	27.08	*Leifsonia shinshuensis* DB 102	NR_043663	99
*Bacillus megaterium* 76-Y149	KU647269	4.78	59.70	*Bacillus* sp. BSp-2	KF835394	99
*Streptomyces* sp. 77-Y1410	KU647270	5.25	34.41	*Streptomyces* sp. N4-145	EF063495	99

### Growth analysis of representative iPSB strains

The four iPSB strains with the highest P-solubilizing capacity were used as representatives for further analysis (*Bacillus megaterium* Y99 was stored in China Center for Type Culture Collection, CCTCC, No. CCTCC AB 2017149). The representative strains were inoculated into 100 mL of liquid modified PVK and incubated at 30 °C for 168 h. The solubilized-P concentration and organic acid production were measured at 12, 24, 48, 96 and 168 h. The solubilized-P concentration was determined by the molybdate-blue method as described above. The P solubilizing percentage (%) was calculated as follows,

$${\text{P}}\;{\text{solubilizing}}\;{\text{percentage}}\;{ = }\;\frac{{P_{free} }}{{P_{total} }}$$

P_free_ indicated the free solubilizing phosphate concentration (µg mL^−1^) in the supernatant of liquid medium, P_total_ indicated the total P concentration (µg mL^−1^) in the liquid medium. The amounts of the organic acids, including lactic, acetic, propionic, gluconic, succinic, oxalic and citric acids, were determined using ion chromatography (ICS-3000, Dionex, USA) as previously described (Hu et al. 2009). The reference standards of the corresponding sodium salts of these organic acids were chromatographically pure (Sigma-Aldrich, Shanghai, China).

### Statistical analyses

Figures were generated using Microsoft Office 365. The sequences were aligned and the phylogenetic trees were constructed and annotated using Clustal X 2.0 (Larkin et al. 2007), MEGA 6.0 (Tamura et al. 2013) and iTOL v3 (Letunic and Bork 2016), respectively. The correlations and variance analyses (ANOVAs) used IBM SPSS Statistics 21.

## Results

### Rapid screening of the iPSB strains

**Fig. 2:**
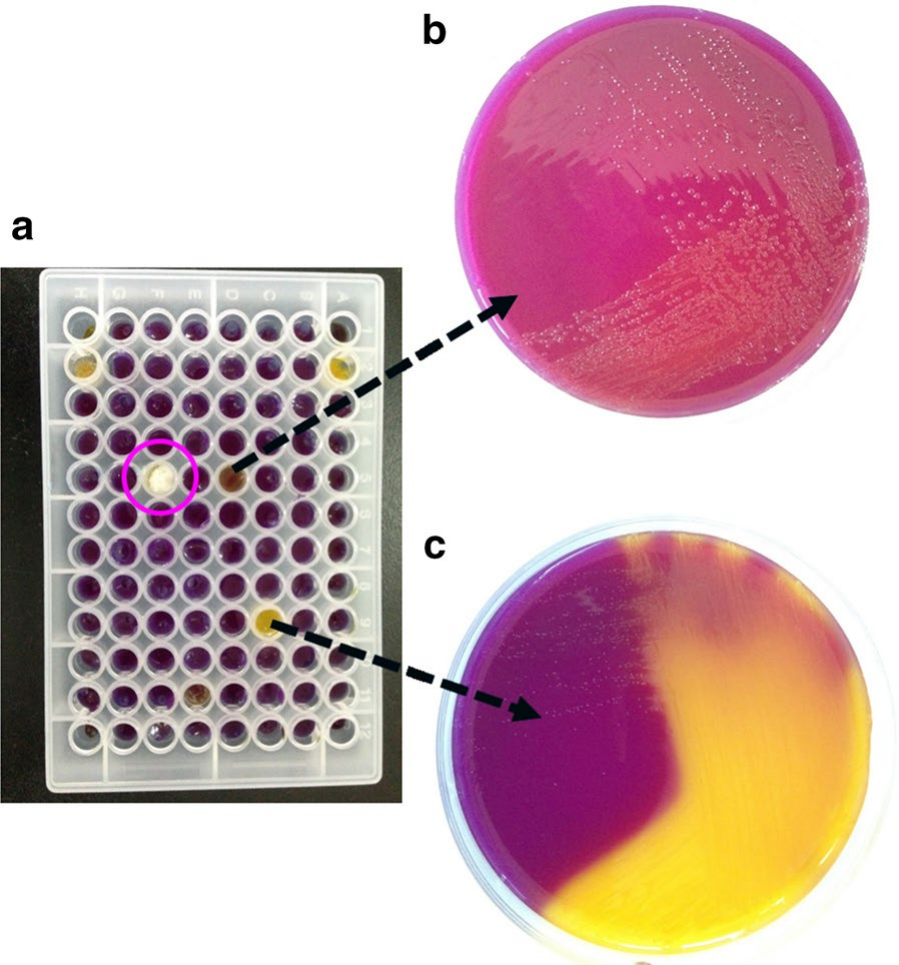
Photographs of **a** a 96-well iPSB screening microplate containing PVK media, **b** an uncertain strain and **c** an iPSB strain cultivated on a PVK medium plate with color change. The purple circle indicates that fungal growth was confined to a single well

Six 96-well microplates (576 wells in total) with modified PVK were used for screening the two soil samples for iPSBs (Fig. 2a). We isolated 39 and 35 iPSB strains from the Hailun and Yingtan soils, respectively, and two uncertain bacterial strains were obtained from the Hailun soil. The uncertain strains were transferred to 200-mm PVK medium plates (Fig. 2b), where they survived and produced slight color changes and clearance zones. We thus classified these two strains as iPSB strains. The other iPSB strains produced obvious color changes after inoculation (Fig. 2c). Hence, a total of 76 iPSB strains were obtained with an average screening efficiency of 13.19 ± 1.47%. Only one bacterial strain survived in each microplate well, which was confirmed by streaking onto 200-mm PVK medium plates. Fungal contamination was well confined to single wells (Fig. 2a, purple circle).

### Phylogenetic identification of iPSB strains

**Fig. 3:**
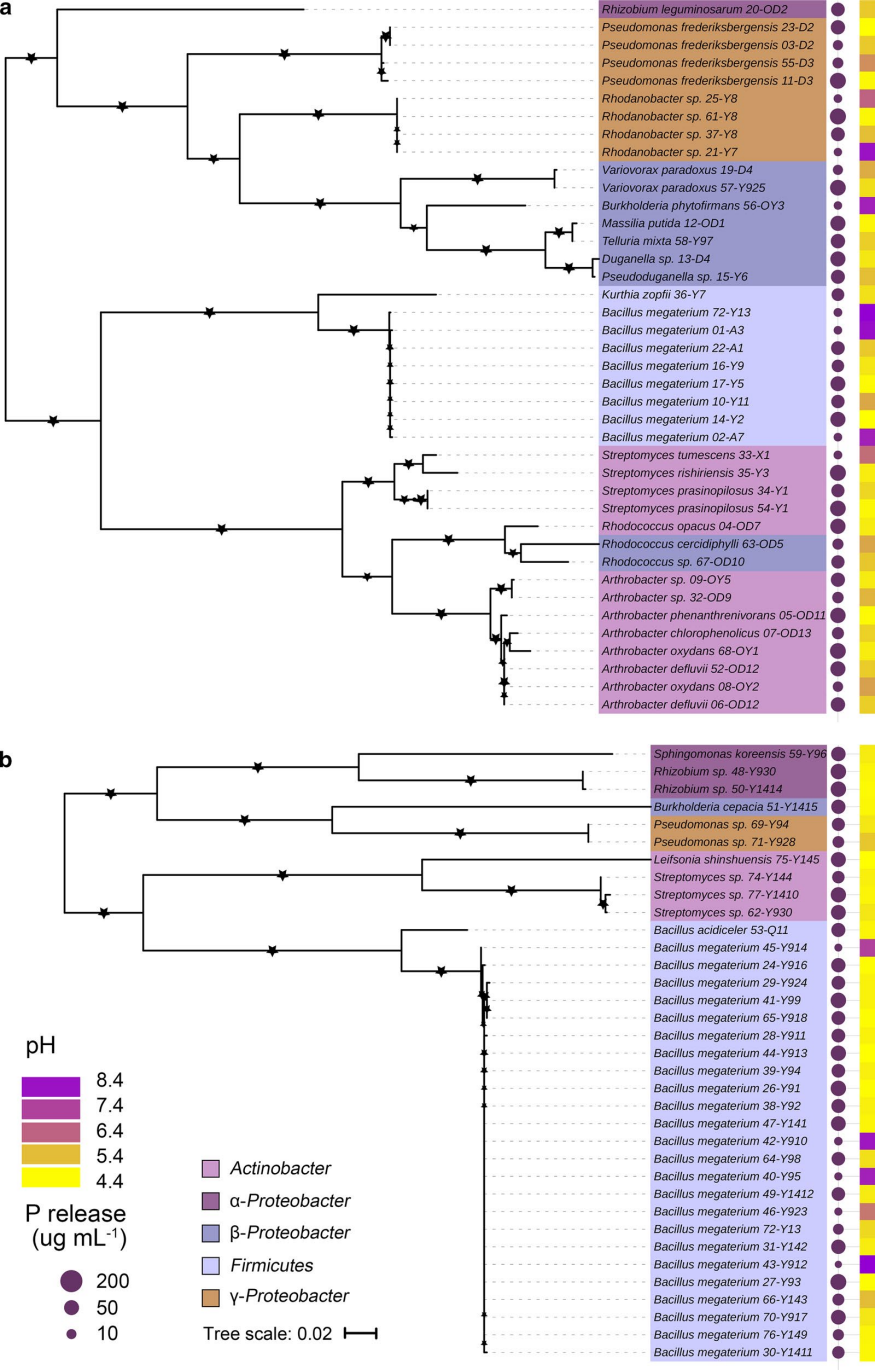
Phylogenic trees of 76 iPSB strains from **a** Hailun and **b** Yingtan soil based on their 16S rRNA sequences. Maximum likelihood was used to construct the trees with bootstrapping (1000 replicates). Bootstrap percentages > 50% are marked with stars. The scale bar indicates 0.02 accumulated changes per nucleotide position

The maximum-likelihood phylogenetic trees based on 16S rRNA sequences from the 76 iPSB strains are presented in Fig. 3. Five phyla were identified: *Actinobacteria*, *Firmicutes* and α-, β- and γ-*Proteobacteria*. *Bacillus megaterium* was the most dominant species (32 isolates). The other 44 isolates were eight *Arthrobacter* sp., seven *Streptomyces* sp., seven *Pseudomonas* sp., four *Rhodanobacter* sp., three *Rhizobium* sp., three *Rhodococcus* sp., two *Burkholderia* sp., two *Variovorax paradoxus* and single isolate of *B. acidceler*, *Duganella* sp., *Kurthia zopfii*, *Leifsonia shinshuensis*, *Massilia* sp., *Pseudoduganella* sp., *Sphingomonas koreensis* and *Tellura mixta*. The closest phylogenic reference strains with their similarities are listed in Table 2. Interestingly, *K. zopfii* 36-Y7 was only 97% similar to the closest strain based on the reference sequence from the GenBank database.

### Biochemical characterization of the iPSB strains

**Fig. 4:**
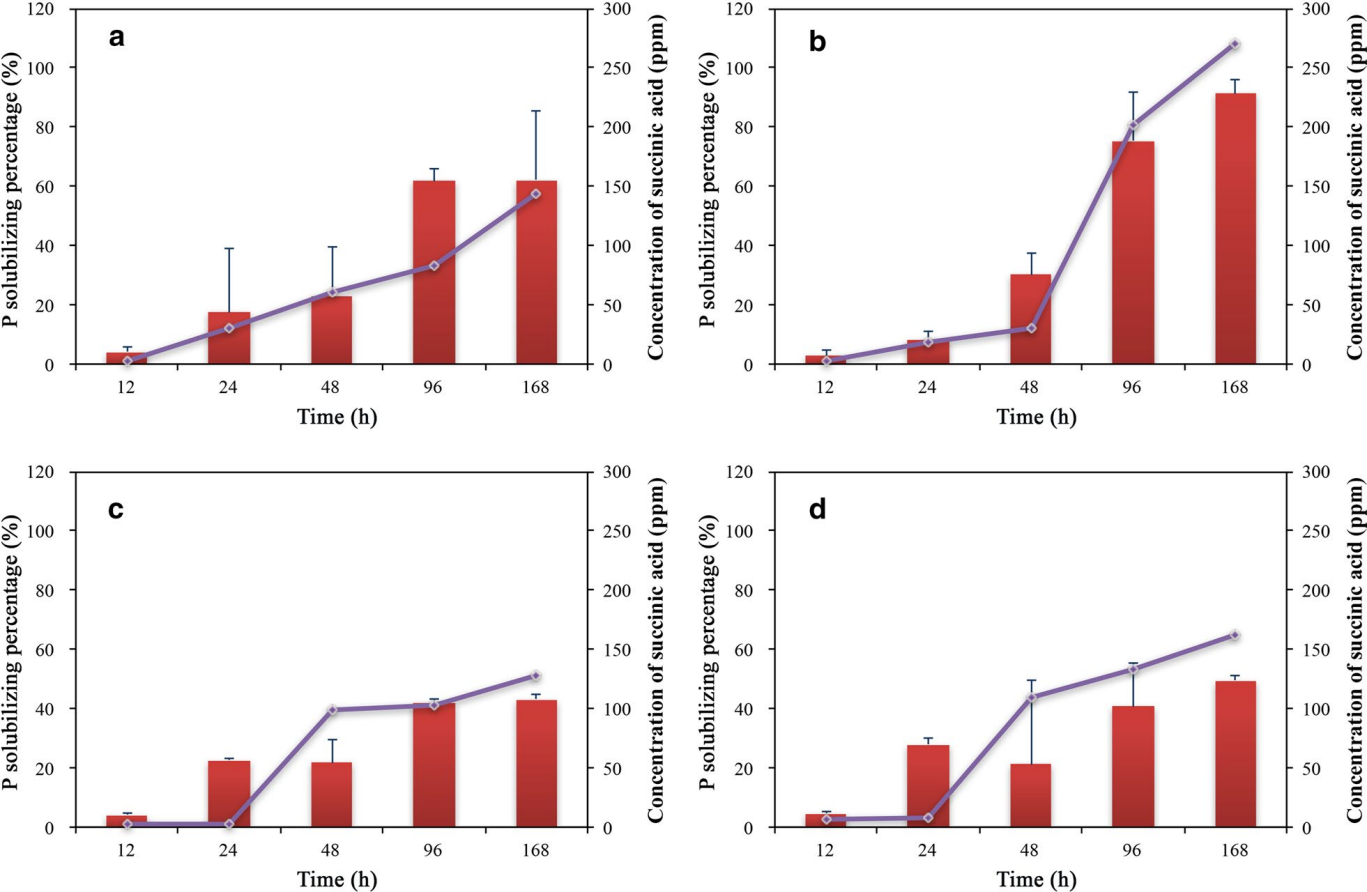
The P-solubilizing percentages (red bars) and succinic acid production (purple lines) for the four representative iPSB strains over 168 h of growth. **a** *B. megaterium* Y1412, **b** *B. megaterium* Y99, **c** *B. megaterium* Y95 and **d** *B. megaterium* Y924. Each value represents the mean of three replicates ± standard deviation

**Fig. 5:**
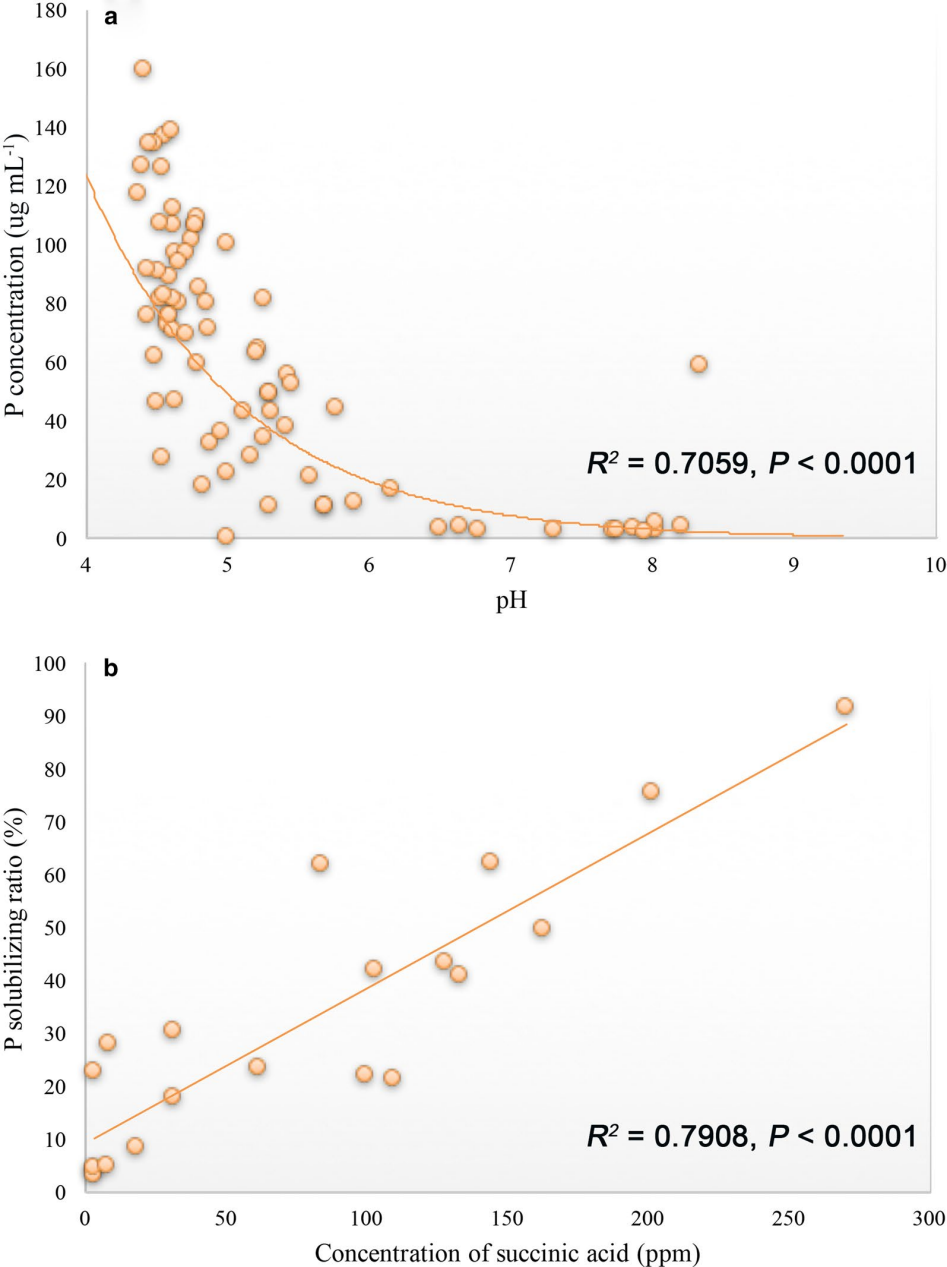
Regression analysis of **a** the correlation between pH and P release for all iPSB strains (regression function: y = 4926.1e^−0.922x^) and **b** the correlation between the concentration of succinic acid and the P-solubilizing percentage for the four representative iPSB strains (regression function: y = 0.2925x + 9.3694)

Details of the medium pH and soluble-P concentration after 72 h incubation are shown in Fig. 3 and Table 2. The pH decreased as the soluble-P concentration increased in the liquid medium. The pH for *B. megaterium* 27-Y93 decreased from an initial 7.0 to 4.37 after 72 h. Interestingly, the pH for *Arthrobacter defluvii* 06-OD12, *Streptomyces tumescens* 33-X1, *Rhizobium* sp. 48-Y930, *Rhizobium* sp. 50-Y1414, *Burkholderia phytofirmans* 56-OY3 and *Streptomyces flaveolus* 60-OD3 increased to 8.34, 8.02, 7.86, 8.02, 8.20 and 7.95, respectively. The soluble-P concentration ranged from 2.03 to 159.48 μg mL^−1^. Neither soluble P nor a decrease in pH was detected in the control treatment. The soluble-P concentration was highest for *B. megaterium* Y99 (159.48 μg mL^−1^), followed by *B. megaterium* Y1412 (138.68 μg mL^−1^), *B. megaterium* Y924 (136.83 μg mL^−1^) and *B. megaterium* Y95 (134.49 μg mL^−1^), and their medium pH decreased to about 4.5. These four strains were further analyzed as representative iPSB strains (Fig. 4a–d). Medium pH was significantly negatively correlated with soluble-P concentration (*P* < 000.1) (Fig. 5a).

### Growth analysis of the four representative iPSB strains

The four representative iPSB strains were incubated with PVK at 30 °C for 168 h. The organic acids were identified by ion chromatography (Table 3). Only three of the seven organic acids (succinic, oxalic and citric) were detected. Among these, the concentration of succinic acid peaked at 209 ppm, which was significantly higher than the concentrations of the other organic acids (*P* < 0.05).

**Table 3:** Amounts of organic acids secreted by the four reference iPSB strains after incubation for 168 h

iPSB strain	Organic acid (ppm)						
	Lactic	Acetic	Propionic	Gluconic	Succinic	Oxalic	Citric
*B. megaterium* Y95	ND	ND	ND	ND	202.30 ± 105.27	6.66 ± 1.22	2.33 ± 0.89
*B. megaterium* Y99	ND	ND	ND	ND	196.60 ± 99.77	6.53 ± 0.06	0.24 ± 0.02
*B. megaterium* Y924	ND	ND	ND	ND	208.88 ± 91.90	5.21 ± 2.10	4.12 ± 0.51
*B. megaterium* Y1412	ND	ND	ND	ND	142.21 ± 27.42	6.23 ± 0.83	2.37 ± 1.67

*ND* not detected

We further analyzed the relationship between the soluble-P and succinic acid concentrations for these four strains (Fig. 4). Both concentrations increased with bacterial growth. A linear regression analysis indicated that the amount of soluble P was significantly positively correlated with the release of succinic acid (*P* < 0.001) (Fig. 5b).

## Discussion

Current agricultural practices tend to use sustainable technology, including the use of biofertilizers with multiple functions, to achieve high crop yields, which requires high soluble-P concentrations. iPSBs thus play an essential role in releasing P from soil-fixation systems and in preserving enough free phosphate in the rhizosphere for plant uptake and growth. iPSB inoculation can promote plant growth (Kaur and Reddy 2015; Yu et al. 2012), so identifying highly efficient iPSB strains thus becomes important. Our rapid screening using 96-well microplates provided an efficient method for simultaneously isolating numerous iPSB strains. The method isolated 76 positive iPSB strains within 72 h from two soils with three replicates. Each well has limited space, so only a single bacterial strain may survive if the sample is sufficiently diluted. Each well in our study contained a sole iPSB strain with a 10^5^ dilution of soil sample from two remote sites with different chemical properties, which was verified by incubation on 200-mm medium plates (Fig. 2), suggesting that further purification by a second or third colony transfer may not be needed. Positive iPSB strains were also easy to observe and count using bromocresol purple as an indicator.

Wells without bacterial growth and a color change were deemed to be negative, and yellow wells (definitely with bacterial growth) were deemed to be positive. Only the uncertain or ambiguous wells may need further confirmation. Two slightly yellow wells in our study were ultimately demonstrated to be positive iPSB strains with P-solubilizing ability. Our method can also effectively prevent the spread of fungal contamination. Most screening media contain glucose or other carbohydrates as carbon sources, so heterologous fungal spores can easily grow or be initially introduced in the diluted sample. Once a single fungal colony appears, it can promptly spread throughout the medium plate and interfere with screening. The first screening of iPSBs from soil samples also may need more than 72 h of incubation, so fungal survival is likely. Fungal spores in our method (Fig. 2a, purple circle), however, were confined to only one well instead of spreading and affecting surrounding wells. The rapid screening with 96-well microplates was demonstrated to be an efficient and effective way to isolate iPSB strains.

The identification of iPSB strains based on 16S rRNA sequences illustrated the phylogenetic structure of the potential iPSB communities in the two soils. *B. megaterium* contributed > 30% to the total iPSB population, which was also most frequently found in the Yingtan soil with an acidic pH (Fig. 3). *Bacillus* is abundant in various types of soil and has a strong P-solubilizing ability in cropland (Chen et al. 2006; Karagöz et al. 2012; Oliveira et al. 2009; Xuan et al. 2011). The next two most common genera, *Arthrobacter* and *Streptomyces*, which belong to *Actinobacteria*, are common in alkaline soils where considerable P is released (Aislabie et al. 2006; Gopalakrishnan et al. 2011; Xiong et al. 2012). *Pseudomonas* is a known iPSB genus and has been well studied due to genetic evidence of inorganic-P solubilization (Babu-Khan et al. 1995; Kwak et al. 2015; Umezawa et al. 2015). Other strains rarely act as iPSBs but can promote plant growth in various types of soils (Richardson et al. 2009; Rodríguez and Fraga 1999; Zhao et al. 2014).

In our study, *B. megaterium* was observed to solubilize more P than the other genera, including *Streptomyces*, *Arthrobacter* and *Pseudomonas*. The presence of *B. megaterium* in the rhizosphere could thus benefit plant P assimilation. *B. megaterium* isolates Y95, Y99, Y924 and Y1412 release > 130 μg mL^−1^ soluble P when cultured in liquid PVK. Lactic and propionic acids were reported to be the two most common organic acids secreted by *B. megaterium* in a previous study (Chen et al. 2006), but we did not observe any ion-chromatographic peaks in the corresponding retention times of these two organic acids (Table 3). Gluconic acid has also been considered an important organic acid for P release (Rodríguez et al. 2006), but we did not detect this acid for any of the four representative iPSB strains. Of the three secreted organic acids, citric and oxalic acids are efficient P-solubilizing acids (Bolan et al. 1994), but their amounts released by these four strains were relatively low and may not be sufficient to liberate much P. The dominant succinic acid was not a common organic acid for P release, but the amounts secreted were strongly correlated with P release for the four representative strains over time (Fig. 4). Succinic acid concentration was strongly, positively and linearly correlated with P release (Fig. 5a) (*R*^2^ = 0.7908, *P* < 0.001), suggesting that succinic acid was the main solubilizing acid secreted by *B. megaterium*.

Environmental pH and organic acid secretion were two common factors accounting for the mobilization of available P. pH is usually negatively correlated with the release of P (Chen et al. 2006; Rodríguez and Fraga 1999). The pH of the medium for some of the iPSBs in our study, however, was alkaline (Table 2), and the iPSBs likely had the ability to release P because they survived well at a high cell density (data not shown). The regression analysis of medium pH and P release (Fig. 5a) indicated a negative but not linear correlation. The pH may only sharply decrease with an increase in P release at acidic pHs, suggesting that soil pH may not be a universal factor to account for microbial P solubilization by iPSB stains.

We demonstrated the efficiency and effectiveness of this rapid iPSB screening method using 96-well microplates. This method is rapid and easy to manipulate and observe and can prevent the spread of fungal growth. *B. megaterium* was the main iPSB strain but released mostly succinic acid rather than other common organic acids for P solubilization. Our study may be useful for mechanistic study of microbial inorganic-P solubilization.

## Abbreviations

P: phosphorus
iPSBs: inorganic phosphate solubilizing bacteria
Olsen P: available P
PVK: Pikovskaya medium
NCBI: National Center for Biotechnology Information
ANOVA: correlations and variance analyses
